# Glypican-3-Targeted Alpha Particle Therapy for Hepatocellular Carcinoma

**DOI:** 10.3390/molecules26010004

**Published:** 2020-12-22

**Authors:** Meghan M. Bell, Nicholas T. Gutsche, A. Paden King, Kwamena E. Baidoo, Olivia J. Kelada, Peter L. Choyke, Freddy E. Escorcia

**Affiliations:** 1 Molecular Imaging Program, National Cancer Institute, National Institutes of Health, Bethesda, MD 20814, USA; meghan.bell@nih.gov (M.M.B.); nick.gutsche@nih.gov (N.T.G.); paden.king@nih.gov (A.P.K.); baidook@mail.nih.gov (K.E.B.); olivia.kelada@perkinelmer.com (O.J.K.); pchoyke@mail.nih.gov (P.L.C.); 2 In Vivo Imaging, Discovery and Analytics, PerkinElmer Inc., Hopkinton, MA 01748, USA; 3 Radiation Oncology Branch, National Cancer Institute, National Institutes of Health, Bethesda, MD 20814, USA

**Keywords:** targeted alpha particle therapy, hepatocellular carcinoma, glypican-3

## Abstract

Glypican-3 (GPC3) is expressed in 75% of hepatocellular carcinoma (HCC), but not normal liver, making it a promising HCC therapeutic target. GC33 is a full-length humanized monoclonal IgG1 specific to GPC3 that can localize to HCC in vivo. GC33 alone failed to demonstrate therapeutic efficacy when evaluated in patients with HCC; however, we posit that cytotoxic functionalization of the antibody with therapeutic radionuclides, may be warranted. Alpha particles, which are emitted by radioisotopes such as Actinium-225 (Ac-225) exhibit high linear energy transfer and short pathlength that, when targeted to tumors, can effectively kill cancer and limit bystander cytotoxicity. Macropa, an 18-member heterocyclic crown ether, can stably chelate Ac-225 at room temperature. Here, we synthesized and evaluated the efficacy of [^225^Ac]Ac–Macropa–GC33 in mice engrafted with the GPC3-expressing human liver cancer cell line HepG2. Following a pilot dose-finding study, mice (*n* = 10 per group) were treated with (1) PBS, (2) mass-equivalent unmodified GC33, (3) 18.5 kBq [^225^Ac]Ac–Macropa–IgG1 (isotype control), (4) 9.25 kBq [^225^Ac]Ac–Macropa–GC33, and (5) 18.5 kBq [^225^Ac]Ac–Macropa–GC33. While significant toxicity was observed in all groups receiving radioconjugates, the 9.25 kBq [^225^Ac]Ac–Macropa–GC33 group demonstrated a modest survival advantage compared to PBS (*p* = 0.0012) and 18.5 kBq [^225^Ac]Ac–IgG1 (*p* = 0.0412). Hematological analysis demonstrated a marked, rapid reduction in white blood cells in all radioconjugate-treated groups compared to the PBS and unmodified GC33 control groups. Our studies highlight a significant disadvantage of using directly-labeled biomolecules with long blood circulation times for TAT. Strategies to mitigate such treatment toxicity include dose fractionation, pretargeting, and using smaller targeting ligands.

## 1. Introduction

Liver cancer is the second leading cause of cancer-related death worldwide, with a total of 840,000 cases and as many deaths each year [1,2]. Hepatocellular carcinoma (HCC) accounts for approximately 85% of liver cancer cases globally [3]. In the U.S., there are 40,000 new HCC cases diagnosed each year, with an average 5 year survival of 19.6% [4]. Moreover, HCC incidence and mortality are increasing, with significant contribution from obesity-related non-alcoholic fatty liver disease (NAFLD), also referred to as metabolic-associated fatty liver disease (MAFLD) [5,6].

The majority of patients with HCC present with advanced disease [4]. Until recently, first-line treatment options have included tyrosine kinase inhibitors sorafenib and lenvatinib [7]. In the IMbrave 150 phase 3 clinical trial of treatment-naïve patients with HCC comparing sorafenib to combined atezolizumab (anti-PD-L1 antibody) and bevacizumab (anti-VEGF antibody), the experimental arm showed a clear survival benefit. These results led to FDA approval of the combination, offering a new first-line treatment option for patients with unresectable HCC [8]. However, while immunotherapy combined with anti-angiogenic agents offers a new modality that can help improve the outcomes of patients with HCC, more work to identify and exploit the unique vulnerabilities of HCC is needed. For example, in the era of precision oncology, no HCC-targeted treatments currently exist as part of the standard of care. 

The HCC-selective target, glypican-3 (GPC3), is a known pathological marker of HCC and is overexpressed in approximately 75% of HCC [9,10,11,12]. GC33 (a.k.a. codrituzumab) is a humanized monoclonal IgG1 specific for GPC3, which has been evaluated in a phase II clinical trial in patients with advanced HCC who had previously progressed on sorafenib. Surprisingly, despite having subnanomolar affinity for GPC3, demonstrating excellent in vivo target binding and therapeutic efficacy in preclinical studies [5,13,14], the effect of GC33 was not different from placebo with respect to progression-free and overall survival [15]. These findings show that, for GC33, antibody-dependent cellular cytotoxicity is not sufficient for an anti-tumor effect in patients and suggest that a cytotoxic payload may be needed to maximize its therapeutic effect.

We posited that coupling GC33 to an alpha particle-emitting radionuclide could enhance therapeutic effect in a preclinical model of HCC. Alpha particles inherently deposit a high amount of ionizing radiation over a distance on the order of cellular diameters. If localized to tumors, alpha particles can cause irreparable double-strand DNA breaks while minimizing dose to neighboring healthy tissues. Actinium-225 is a clinically relevant radionuclide that has been used in patients to deliver alpha particles to leukemia [16], prostate [17,18], and neuroendocrine cancers [19].

Conventionally, the metal chelator DOTA is conjugated to tumor-targeted ligands (e.g., peptides or antibodies) to create the targeted alpha particle therapeutic (TAT) agent. Chelation of Ac-225 by DOTA requires temperatures in the 60–80 °C range, which can denature many biomolecules, including antibodies. Recently, Thiele et al. showed that Macropa, an 18-member heterocyclic crown ether, can stably chelate Ac-225 at room temperature [20]. Here, we synthesized and characterized a [^225^Ac]Ac–Macropa–GC33 radioconjugate and assessed its therapeutic effect and toxicity in mice using xenografts of the liver cancer cell line HepG2. 

## 2. Results

### 2.1. [^225^-Ac]Ac–Macropa–GC33 is Stable for up to 336 h in Human Serum

**Figure 1:**
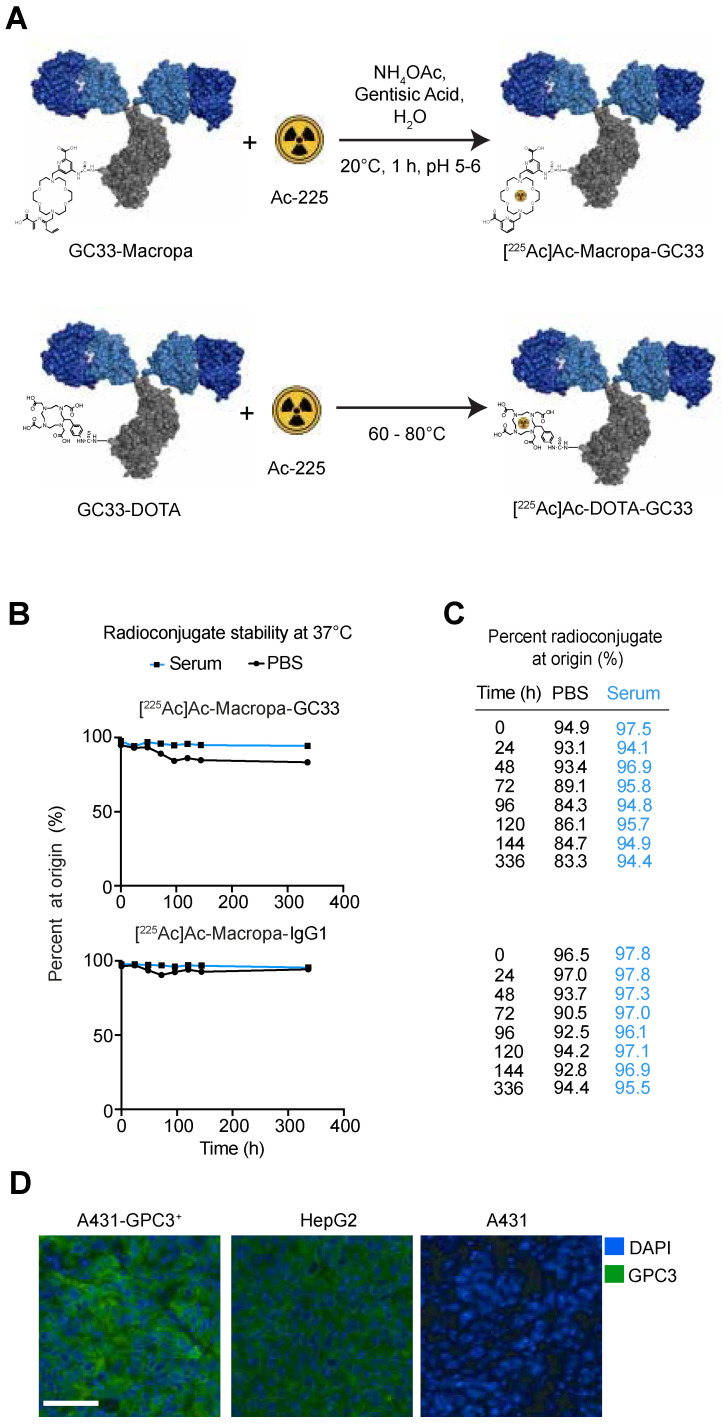
^225^Ac-labeled Macropa radioimmunoconjugates are stable in serum over time. (**A**). Schema for one-step Ac-225 labeling of antibody radioconjugates underscores that, unlike DOTA, Macropa chelation can occur at room temperature. (**B**,**C**). [^225^Ac]Ac–Macropa–GC33 and [^225^Ac]Ac–Macropa–IgG1 are stable in human serum and PBS at 37 °C up to 336 h after radiolabeling as measured by thin-layer chromatography and percent of activity at origin. (**D**). Immunofluorescence staining for GPC3 with fluorescently labeled GC33 (green) and DAPI (blue) counterstain of tumors derived from A431-GPC3^+^, HepG2, and A431 (scale bar 50 µm).

Mass spectrometry demonstrated an average of 10 (range 7–12) Macropa moieties (∆590.23 g/mol) per GC33 (148,184 g/mol) (Figure S1A). Radiochemical yields were typically >80% and radiochemical purity was ≥98% for all radioconjugates (Figure S1B,C). Aliquots of [^225^Ac]Ac–Macropa–GC33 and [^225^-Ac]Ac–Macropa–IgG1 were taken and analyzed at various time points post-conjugation using radio-iTLC to determine stability of each purified conjugate. Over time, [^225^Ac]Ac–Macropa–GC33 demonstrates good stability in both PBS (83.3% at 336 h) and human serum (94.4% at 336 h) (Figure 1B,C). Similarly, [^225^-Ac]Ac–Macropa–IgG1 was stable in both PBS (94.4% at 336 h) and human serum (95.5% at 336 h). These observations suggest that both radioconjugates may remain stable in vivo for up to 336 h (14 days) post-injection (p.i.).

### 2.2. Biodistribution Studies Show Preferential Tumor Localization of [^225^Ac]Ac–Macropa–GC33 Compared to [^225^Ac]Ac–Macropa–IgG1

**Figure 2:**
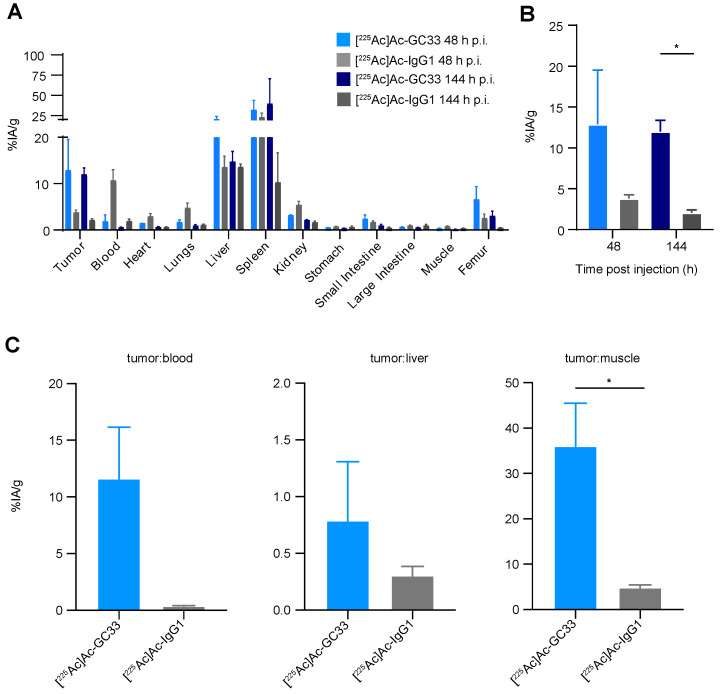
[^225^Ac]Ac–GC33 specifically localizes to HepG2 tumors. (**A**). Biodistribution studies show notable accumulation of either radioconjugate in spleen, liver, and tumor tissues in the HepG2 tumor model. (**B**). [^225^Ac]Ac–GC33 demonstrates preferential tumor uptake in the HepG2 tumor model at 144 h p.i. compared to [^225^Ac]Ac–IgG1 (*p* = 0.0120), but not at 48 h p.i. (*p* = 0.2415) (**C**). There are quantitatively higher tumor:blood and tumor:liver ratios in animals receiving [^225^Ac]Ac–GC33, with tumor:muscle ratios significantly higher for animals given [^225^Ac]Ac–GC33 compared to [^225^Ac]Ac–IgG1 (*p* = 0.0304). Error bars represent the standard error of the mean (SEM). Statistical significance was defined by *p* < 0.05 (*).

To determine the biodistribution of the [^225^Ac]Ac–Macropa–GC33 and [^225^Ac]Ac–Macropa–IgG1 radioconjugates, we used a HepG2 subcutaneous flank model and administered 9.25 kBq of each conjugate. [^225^Ac]Ac–Macropa–GC33 exhibited higher specific accumulation in HepG2 tumors at 48 h p.i. than [^225^Ac]Ac–Macropa–IgG1, (mean ± standard error to the mean (SEM)) percent injected activity per gram (%IA/g) of 12.9 ± 6.6 versus 3.78 ± 0.48) (Figure 2A,B). Similar results were observed at 144 h p.i., with %IA/g values of 11.98 ± 1.4 versus 2.08 ± 0.32 (*p* = 0.0120) for [^225^Ac]Ac–Macropa–GC33 and [^225^Ac]Ac–Macropa–IgG1, respectively (Figure 2A,B). Tumor:blood, tumor:liver, and tumor:muscle ratios were favorable for the GC33-based radioconjugate compared to control. However, the tumor:liver values suggest substantial non-specific liver accumulation.

### 2.3. Radioimmunotherapy with [^225^Ac]Ac–Macropa–GC33 Shows Tumor Reduction Compared to [^225^Ac]Ac–Macropa–IgG1

To determine the optimal dose for [^225^Ac]Ac–Macropa–GC33, a pilot study was completed in a HepG2 subcutaneous flank model to test multiple administered activities of this radioconjugate. Treatment with 18.5 kBq of [^225^Ac]Ac–Macropa–GC33 was found to cause tumor growth delay and was well tolerated. Therefore, we used 18.5 kBq as the highest amount of activity administered in our subsequent therapy study. We also tested 9.25 kBq in an attempt to mitigate toxicity while preserving therapeutic effect.

**Figure 3:**
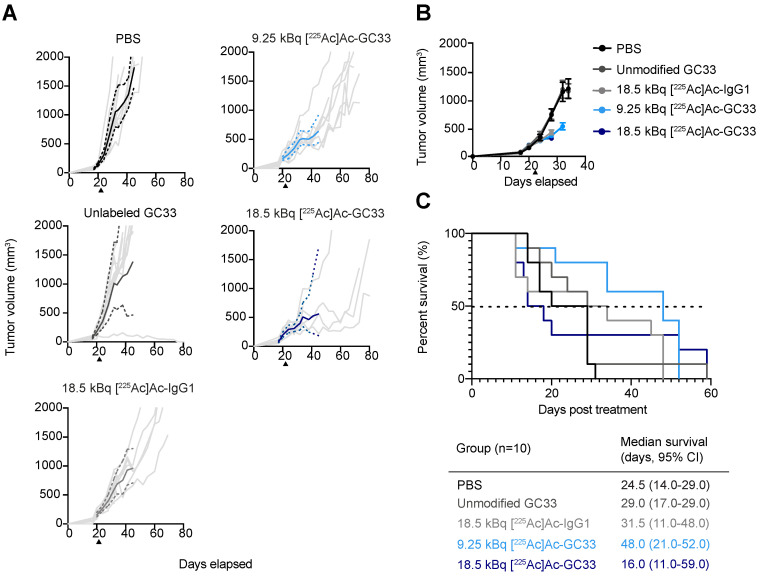
Local control and survival of tumor-bearing mice. (**A**). Spider plots showing the tumor volume of individual mice (light grey), with the mean (bold solid lines) and 95% confidence interval (CI) (dashed lines) of treatment groups over time. (**B**). Mean tumor volumes ± SEM, and (**C**). Kaplan–Meier survival plot. Log-rank test shows survival is significantly higher for the cohort treated with 9.25 kBq [^225^Ac]Ac–Macropa–GC33 compared to PBS control (*p* = 0.0012) and 18.5 kBq [^225^Ac]Ac–Macropa–IgG1 (*p* = 0.0412).

To evaluate the therapeutic efficacy of [^225^Ac]Ac–Macropa–GC33, we recorded tumor volumes and assessed survival of all groups. PBS and unmodified GC33 controls approached 2000 mm^3^ as early as 14 days post-injection, necessitating sacrifice. Compared to control cohorts treated with PBS and unmodified GC33, a modest tumor growth delay was observed for the [^225^Ac]Ac–Macropa–IgG1 cohort. This observation signals therapeutic benefit of the radiolabeled non-specific control, likely due to the enhanced permeability and retention effect (EPR) [21]. Tumor reduction was also observed when comparing spider plots of tumor volumes for the 9.25 and 18.5 kBq [^225^Ac]Ac–Macropa–GC33 cohorts to the [^225^Ac]Ac–Macropa–IgG1 cohort. This dampening of tumor growth is evident when comparing the geometric means of the cohorts treated with either radioconjugate (Figure 3A, solid lines).

Mean tumor volumes for each group were plotted until the first recorded death in each group, resulting in an inability to assess long-term tumor reduction effect for Ac-225-treated animals (Figure 3B). Accordingly, to determine if tumor growth delay was significant for the groups treated with either radioconjugate, we performed a Chi-square analysis of the number of animals bearing tumor volumes exceeding 500 mm^3^ (at least 2-fold larger than initial tumor volume) at 7 days p.i., the latest time point that all animals in each group were alive (Figure S2B). The number of animals exceeding 500 mm^3^ in tumor volume was significantly higher in PBS (*p* = 0.0010), unmodified GC33 (*p* < 0.0001), and 18.5 kBq [^225^Ac]Ac–Macropa–IgG1 (*p* = 0.0253) when compared to 18.5 kBq [^225^Ac]Ac–Macropa–GC33. There was also a greater number of animals reaching 500 mm^3^ in tumor volume in the PBS cohort compared to the 9.25 kBq [^225^Ac]Ac–Macropa–GC33 group (*p* = 0.0062). A Student’s *t*-test was performed to compare the average tumor volumes between treatment groups at 7 days p.i. Animals that received 18.5 kBq [^225^Ac]Ac–Macropa–IgG1 had a greater average tumor burden (425.2 mm^3^, 95% CI: 329.1–521.2 mm^3^) than those that received 18.5 kBq [^225^Ac]Ac–Macropa–GC33 (318.0 mm^3^, 95% CI: 258.7–377.3 mm^3^) (*p* = 0.0456).

### 2.4. Lower Administered Activity of [^225^Ac]Ac–Macropa–GC33 Shows Improved Survival Compared to Higher Administered Activity of IgG1 Control

Unanticipated toxicity necessitating sacrifice of animals in the radioconjugate-treated groups had a clear impact on survival. The median survival was 31.5 d (95% CI: 11.0–48.0 d) p.i. for the 18.5 kBq [^225^Ac]Ac–Macropa–IgG1 cohort, and 16.0 d (95% CI: 11.0–59.0 d) for the 18.5 kBq [^225^Ac]Ac–Macropa–GC33 cohort (Figure 3C). Mice in the 9.25 kBq [^225^Ac]Ac–Macropa–GC33 group exhibited less toxicity compared to the higher administered activity and had a median survival of 48.0 d (95% CI: 21.0–52.0 d) p.i. Log-rank analysis showed that survival was significantly higher for the 9.25 kBq [^225^Ac]Ac–Macropa–GC33 cohort compared to the 18.5 kBq [^225^Ac]Ac–Macropa–IgG1 (*p* = 0.0412) and PBS cohorts (*p* = 0.0012) (Figure 3C).

### 2.5. Partial Recovery of Hematologic Toxicity in the 9.25 kBq [^225^Ac]Ac–Macropa–GC33 Cohort

In addition to monitoring weight loss, a complete blood count was performed at regular intervals following treatment to assess hematologic toxicity. While animals in the PBS and unmodified GC33 groups maintained average weights well above 90% of their starting weight, groups receiving any radioconjugate exhibited significant weight loss early in the study. At 7 days p.i., the weights of the 18.5 kBq [^225^Ac]Ac–Macropa–IgG1 and 18.5 kBq [^225^Ac]Ac–Macropa–GC33 groups fell to 87.3 ± 1.3% and 83.0 ± 3.2% (mean ± SEM) of their starting body weight, respectively. Further, animals receiving 9.25 kBq [^225^Ac]Ac–Macropa–GC33 experienced less dramatic weight loss, dropping to to 90.4 ± 1.4% of their starting weight at 7 days p.i. (Figure S2A). However, animals that were treated with either radioconjugate never returned to their initial body weight, suggesting persistent toxicity for the duration of the study.

**Figure 4:**
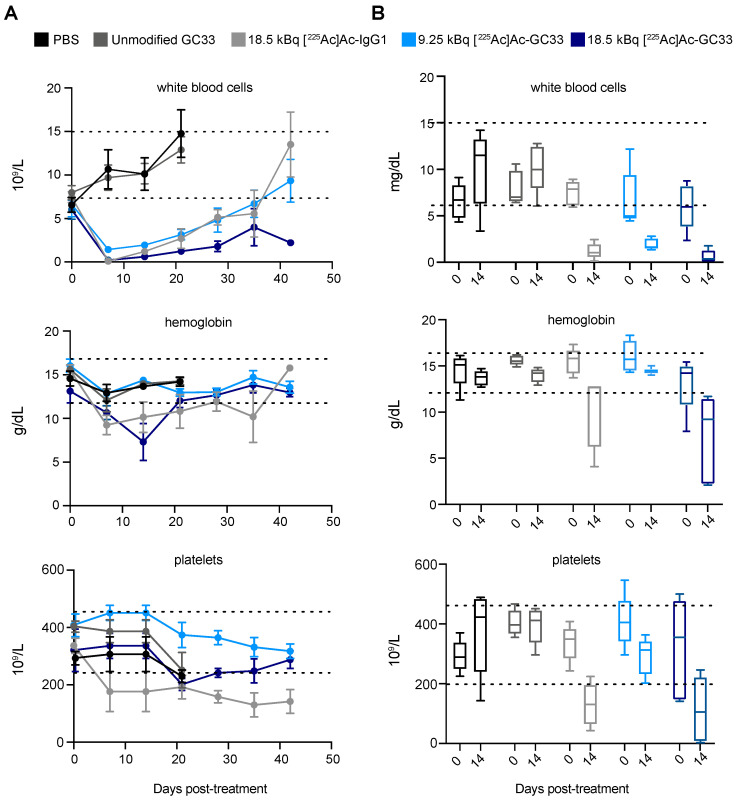
Complete blood count (CBC) shows acute hematological toxicity and gradual recovery following treatment with ^225^Ac-labeled radioconjugates. (**A**). White blood cell count, hemoglobin, and platelets were measured as part of CBC analysis of whole blood samples and plotted over time (mean ± SEM). Counts for all parameters drop dramatically 7 days p.i. for groups treated with either radioconjugate, followed by eventual recovery of majority of parameters to within normal range denoted by dotted lines (*n* = 5). (**B**). Measurement of white blood cells, hemoglobin, and platelets from day 0 and day 14 p.i. were plotted on box-and-whisker plots and compared. Unlike other radioconjugate-treated groups, there was a less dramatic decrease in white blood cell count for the 9.25 kBq group hemoglobin and platelet counts remained in normal range for the duration of the study. Error bars represent the minimum and maximum values observed, the length of the box represents the range from the 25th to 75th percentile, and the horizontal line within the box represents the median. Dotted lines represent the normal range of values.

Acute bone marrow toxicity was observed for animals treated with any radioconjugate 7 days p.i. as evidenced by a notable drop in white blood cell (WBC) count (Figure 4A). Interestingly, while the WBC count dropped below normal range for all groups receiving any radioconjugate, hemoglobin and platelet values remained within normal ranges in animals treated with 9.25 kBq [^225^Ac]Ac–Macropa–GC33. At 14 days p.i., WBC counts were higher in animals treated with 9.25 kBq of [^225^Ac]Ac–Macropa–GC33 compared to those treated with 18.5 kBq [^225^Ac]Ac–Macropa–GC33 (*p* = 0.0150) (Figure 4B). Notably, while it took 42 days for WBCs to recover in the 9.25 kBq [^225^Ac]Ac–Macropa–GC33- and the 18.5 kBq [^225^Ac]Ac–Macropa–IgG1-treated groups, those of the 18.5 kBq [^225^Ac]Ac–Macropa–GC33 group never recovered.

### 2.6. Liver and Kidney Function Are Stable 

**Figure 5:**
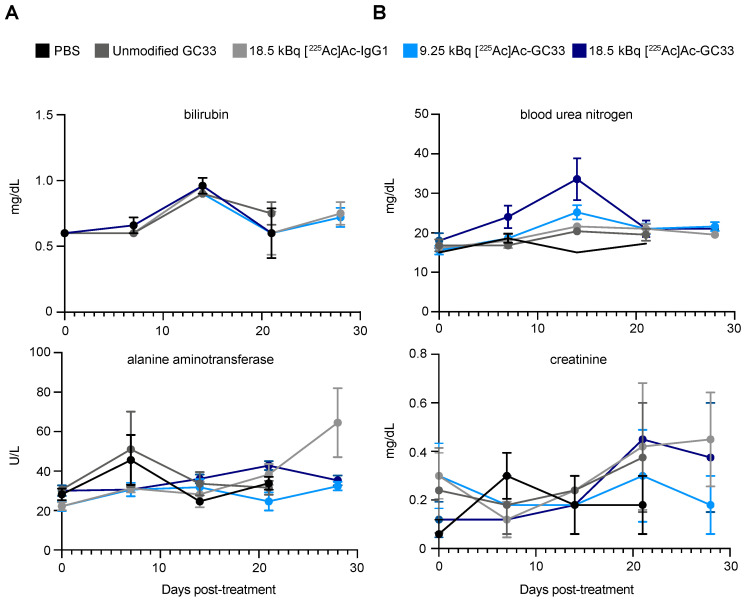
Markers of liver and kidney function are stable following treatment with radioimmunoconjugates. (**A**). Markers of liver damage, bilirubin and alanine aminotransferase. (**B**). Markers of kidney function, blood urea nitrogen and creatinine exhibit minor changes following treatment compared with control-treated groups, suggesting that toxicity to liver and kidneys is limited. Error bars represent the standard error of the mean (SEM).

Basic Metabolic Panel (BMP) analysis of liver function enzymes bilirubin and alanine-aminotransferase showed minimal fluctuations from baseline up to 28 days p.i. (Figure 5A), suggesting minimal hepatotoxicity with any treatment. In addition, no remarkable changes to kidney function as measured by creatinine and blood urea nitrogen were observed, suggesting that nephrotoxicity was minimal.

## 3. Discussion

The GPC3-specific humanized monoclonal IgG1 GC33 (a.k.a. codrituzumab) has previously demonstrated excellent preclinical and clinical localization to HCC-expressing GPC3 in vivo [5,13]. However, in a phase II trial of patients with advanced HCC, when compared to placebo control-treated patients, GC33 did not change progression-free or overall survival [15]. We hypothesize that the putative mechanism of cell kill for GC33, antibody-dependent cellular cytotoxicity, was insufficient to exert a therapeutic benefit in these patients. Given that GC33 has been previously shown to localize to GPC3-expressing HCC and the limited treatment options available, we aimed to label GC33 with a cytotoxic alpha particle-emitting isotope to evaluate its therapeutic efficacy in an animal model of HCC [5,13]. Here, we functionalized GC33 with the Macropa chelate, and labeled the conjugate with Ac-225 to yield [^225^Ac]Ac–Macropa–GC33. Importantly, we selected the Macropa chelate because of its ability to label Ac-225 at room temperature, as opposed to at 60–80 °C as is required for DOTA, which can prevent antibody denaturation during the radiolabeling process (Figure 1A) [20]. We demonstrate that [^225^Ac]Ac–Macropa–GC33 has a modest anti-tumor effect as measured by tumor growth and survival in HepG2 murine xenograft models, though caution is warranted because of significant treatment-related toxicity.

In this study, we tested two administered activities of [^225^Ac]Ac–Macropa–GC33: 18.5 kBq, which was determined to be effective and well tolerated in a pilot study, and 9.25 kBq, a lower amount that was tested to assess whether we might achieve comparable anti-tumor benefit while mitigating potential radiotoxicity. Mice treated with PBS, mass-equivalent unmodified GC33, and 18.5 kBq [^225^Ac]Ac–IgG1 served as controls. While we observed tumor growth delay in all ^225^Ac conjugates, including the IgG1 isotype control, results were more dramatic for animals treated with GC33-derived radioconjugates. This enhanced tumor growth delay in animals treated with either amount of [^225^Ac]Ac–Macropa–GC33 compared to [^225^Ac]Ac–Macropa–IgG1 is supported by biodistribution data confirming the ability of [^225^Ac]Ac–Macropa–GC33 to preferentially target GPC3 in vivo in the HepG2 tumor model. In fact, the tumor uptake was nearly identical to that of a GC33-derived immunoPET agent (~15% IA/g) [13]. Moreover, 9.25 kBq [^225^Ac]Ac–Macropa–GC33 was better tolerated than 18.5 kBq and showed significant improvement in overall survival compared to PBS and unmodified GC33 controls. The fact that we observed substantial toxicity in the 18.5 kBq cohort in this study suggests that this amount of administered activity may be at the steep portion of the toxicity–activity curve. 

Bone marrow suppression was observed within days of treatment for groups given any radioconjugate. These findings likely reflect the long blood half-life of full-length antibodies that, when coupled to alpha particle-emitting radionuclides, can cause non-selective irradiation of the blood pool and hematopoietic progenitors, phenomena that mirrors what is observed in clinical studies using these agents [16]. Furthermore, it is possible that the Fc receptors (FcR) present on macrophages and B-cells, which can bind the Fc of full-length antibodies such as GC33, may have also contributed to a decrease in white blood cell counts, as has been previously described [22]. Given the off-tumor accumulation of our radioconjugate in the liver and spleen, which bear resident macrophages, our findings are consistent FcR-associated toxicity as well. 

Comparing the complete blood counts of animals receiving 9.25 and 18.5 kBq of [^225^Ac]Ac–Macropa–GC33 provides valuable information for mitigating potential radiotoxicity in future studies. Notably, the hemoglobin and platelet counts remained in the normal range for the 9.25 kBq [^225^Ac]Ac–Macropa–GC33 treatment cohort throughout the course of our study, unlike cohorts receiving 18.5 kBq. While these observations include an element of survivor bias, the shorter duration of marrow suppression in animals receiving a lower injected activity of radioconjugate may very well have allowed this group to achieve the modest survival benefit not observed in other groups. Importantly, liver and kidney function appear to have been minimally impacted throughout the treatment course.

There are several limitations to our current study. Given the toxicity observed, optimization of the amount of activity and the administration of our conjugate are needed. Several strategies to address these issues are available, including fractionation [23,24] and pretargeting, which decouples the long blood half-life targeting agent from the cytotoxic cargo [25]. The use of smaller targeting ligands (e.g., antibody fragments, peptides or small molecules), which may have more favorable pharmacokinetics than full-length antibodies, can also be considered. In addition, while the amount of administered activities used here were empirically determined in a pilot study using the same preclinical model, our results clearly indicate that further tuning is required given the toxicity observed. Such dose optimization is critical in this model prior to exploring outcomes in more complex systems (e.g., orthotopic) and for clinical translation. Moreover, we anticipate that employing models to estimate the absorbed dose of TAT agents to tumors and normal tissues may predict therapeutic efficacy and toxicity, which is currently an area of active investigation [26].

## 4. Materials/Methods

### 4.1. Cell Culture

HepG2, a human hepatoblastoma cell line (GPC3^+^), and A431, an epithelioid cancer line, were purchased from ATCC (Manassas, VA, USA) and cultured according to vendor’s instructions. A431-GPC3^+^ was engineered to stably overexpress GPC3 as previously described [27]. Cells were maintained with DMEM media (Life Technologies, Carlsbad, CA, USA) supplemented with 10% FetalPlex (Gemini Bio-Products, Sacramento, CA, USA). Cells tested negative for mycoplasma and were kept at 37 °C and 5% CO_2_ in a humified incubator. Cells were implanted within 15 passages. 

### 4.2. Antibodies

Codrituzumab (GC33), a humanized anti-GPC3 IgG1, was obtained from Chugai Pharmaceutical Co. Ltd. (Tokyo, Japan) and Obinutuzumab (IgG1), a humanized anti-CD20 IgG1, was purchased from the NIH Division of Veterinary Resources and used as an IgG1 isotype control. 

### 4.3. Conjugation of GC33 and IgG1 Antibodies to Macropa-isothiocyanate

The GC33 formulation (40 mg/mL 0.150 mL) or IgG1 formulation (25 mg/mL; 0.5 mL) was buffer exchanged into PBS (pH 8.0), to be spun down (4500 RPM for 50 min at 25 °C) to a concentration of 67 mg/mL and 32 mg/mL, respectively, using 30,000 MWCO Amicon Ultra centrifugal filter units (EMD Millipore, Burlington, MA, USA). The buffer-exchanged GC33 (1.2 mg, 0.018 mL, 8.1 nmol) and IgG1 (2 mg, 0.063 mL, 13.7 nmol) were transferred to 1.5 mL Eppendorf tubes and the pH was adjusted to 9.1 with a solution of Na_2_CO_3_ (0.1 M). A solution of NaHCO_3_ (0.100–0.200 mL, 0.1 M, pH 9.2) was added to the GC33 reaction mix, while NaHCO_3_ buffer (0.115–0.140 mL, 0.1 M, pH 9.2) was also added to the IgG1 mixture. Solutions of Macropa-isothiocyanate(NCS) (courtesy of Wilson lab at Cornell University, MW: 1045.76 g/mol) in 0.1 M NaHCO_3_ (12.45 μL, 121 nmol; 15:1 molar excess over GC33) and (28 μL, 273 nM; 20:1 molar excess over IgG1) were pipetted dropwise into the Eppendorf vials over 3 min with intermittent gentle vortexing. Both reaction mixtures were placed on a thermomixer (Eppendorf, Hamburg, Germany) and shaken at 37 °C for 1–2 h. After incubation, unbound Macropa-NCS was removed with PD-10 desalting columns (GE Healthcare, Piscataway, NJ, USA) using PBS (GC33) or NH_4_OAc buffer (IgG1) (0.15 M, pH 7) as eluate and the product was concentrated in a 30,000 MWCO Amicon Ultra centrifugal filter unit. 

### 4.4. Mass Spectrometry Analysis of GC33–Macropa and IgG1–Macropa Conjugates

Mass spectrometry via an Exactive Plus Extended Mass Range benchtop Orbitrap system with a heated electrospray ionization source (HESI) was used to determine the number of DFO chelators coupled per antibody as previously described [27]. GC33–Macropa conjugates were compared with unmodified GC33 via mass spectrometry to determine the number of moieties covalently bound to GC33. The number of Macropa moieties per GC33 was estimated using the molecular weights of Macropa (590.23 g/mol) and unmodified GC33. Conjugates and unmodified proteins were analyzed on an Exactive Plus EMR Orbitrap system (Thermo Scientific, Waltham, MA, USA). An amount of 1 µg of protein was loaded onto a 4 µM bead size MAbPac RP column (3 × 50 mm, Thermo Scientific) utilizing a Vanquish UHPLC (Thermo Scientific) connected to an Exactive Plus EMR mass spectrometer. The proteins were eluted with a 2 to 100% gradient of 80% acetonitrile with 0.1% formic acid over 8 min with a flow rate of 0.5 mL/min. The EMR was operated with three sequential orbitrap scan methods that each acquired MS1 at 8750 resolution with a maximum injection time of 100 ms and an AGC target of 3e6. Scans looked at the range of 600–10,000 *m/z*. The first segment included no additional solvation energy, and had 3 summed microscans, the second segment used 30 eV additional solvation energy and 5 microscans and the third microscan with 50 eV additional solvation energy used 10 microscans. All data were processed in BioPharma Finder 2.0 using the respect algorithm with a maximum mass deviation of 20 ppm with sliding windows [23]. 

### 4.5. Radiolabeling GC33–Macropa and IgG1–Macropa Conjugates with Ac-225

Gentisic acid (20 µL, 0.1 M) was added to a solution of Ac-225 (7.40–14.8 MBq, Oak Ridge National Laboratories, Oak Ridge, TN, USA) in hydrochloric acid (25 µL, 0.1 M). The mixture was neutralized to pH 5–6 by addition of NH_4_OAc buffer (0.4–0.8 µL, 5 M). This stock solution was portioned between Macropa–IgG1 or Macropa–GC33 (0.4 mg) in NH_4_OAc buffer (0.15 M). The reaction mixture was incubated at room temperature with gentle agitation for 1 h and was quenched by the addition of ethylenediaminetetraacetic acid (EDTA) solution (5 µL, 0.1 M). The radioconjugates were purified using a PD-10 desalting column with PBS as the eluate. Radiochemical yield and purity were determined by radio-iTLC with silica gel-impregnated glass-microfiber paper strips (iTLC-SG, Varian, Lake Forest, CA, USA) using an aqueous solution of EDTA (10 mM, pH 5.5) as the mobile phase. Data were analyzed using an AR-2000 (Eckert-Ziegler, Wilmington, MA, USA) to calculate the percent of total activity at the origin. 

### 4.6. Serum Stability of [^225^Ac]Ac–Macropa–GC33 and [^225^Ac]Ac–Macropa–IgG1

To assess the stability of the purified [^225^Ac]Ac–Macropa–GC33 radioconjugate, the compound was incubated at 37 °C in PBS or human serum. At several time points following incubation, 1 µL aliquots were taken and run on radio-iTLC with silica gel-impregnated glass-microfiber paper strips, using as a mobile phase an aqueous solution of EDTA (10 mM, pH 5.5), which was then analyzed using an AR-2000. Percent of total activity at the origin versus total activity was used to determine the intact radioconjugate.

### 4.7. Murine Subcutaneous Xenograft Models

All procedures were approved by the Institutional Animal Care and Use Committee at the National Institutes of Health under protocol ROB-105. Female athymic homozygous nude mice, strain Crl:NU(NCr)-Foxn1^nu^ (Charles River Laboratories, Wilmington, MA, USA), with ages between 8–10 weeks, were subcutaneously xenografted on the right flank with 1.0–2.0 × 10^6^ HepG2 cells in a 200 µL solution of a 1:1 mixture of Matrigel (Corning, Corning, NY, USA) and cell suspension. Tumors were grown to a range of 100–200 mm^3^ post-implantation prior to beginning treatment. HepG2 cells were selected for their high native expression of GPC3 as shown previously [13]. 

### 4.8. Immunofluorescence

Tumors derived from A431-GPC3^+^, HepG2, and A431 subcutaneously engrafted in female athymic nude mice were excised, rinsed with PBS and submerged in molds containing TissueTek OCT medium (Sakura Fineteck Inc, Torrence, CA, USA) and frozen on dry ice. Consecutive 10 µm sections were cut using a cryostat (Leica Biosystems, Buffalo Grove, IL, USA) from prepared frozen tissue blocks and placed on slides (Azer Scientific Inc., Morgantown, PA, USA). Tissue sections were incubated at room temperature to dry for 30 min and fixed in 100% methanol at −20 °C for 10 min, then left to air dry for 10 min, and rinsed in PBS. Blocking buffer was composed of 0.5% IgG-free BSA (Jackson Labs, Bar Harbor, ME, USA), 2.2% glycine (Sigma-Aldrich, St. Louis, MO, USA), 0.1% Tween-20 (Sigma-Aldrich), 5% donkey serum (Jackson Labs, Bar Harbor, ME, USA) solution in PBS. Slides were blocked with blocking buffer for 30 min at room temperature before being incubated with 10 µg/mL of GC33-AF488 antibody (diluted in blocking buffer) for 1 h at room temperature. Slides were subsequently rinsed in PBS and counterstained with 1:5000 dilution of 4’,6-diamidino-2-phenylindole dihydrochloride (DAPI, Invitrogen, Eugene, OR, USA) in deionized water for 3–5 min at room temperature. Slides were rinsed in deionized water, mounted with Fluoro-gel water-soluble mounting medium (Electron Microscopy Sciences, Hatfield, PA, USA), and cover slips (Thermo Fisher, Portsmouth, NH, USA) were placed. Stained slides were stored in the dark at 4 °C. Fluorescent microscopy images were acquired using a Nikon Ti2 widefield microscope.

### 4.9. Targeted Alpha-Particle Therapy with [^225^Ac]Ac–Macropa–GC33

Once tumors of mice engrafted with HepG2 reached 100–200 mm^3^, animals were randomized into groups (*n* = 10). Groups were treated with: (1) PBS, (2) 3 µg unmodified GC33, (3) 18.5 kBq, 3 µg [^225^Ac]Ac–Macropa–IgG1, and (4) 9.25 kBq, 1.5 µg [^225^Ac]Ac–Macropa–GC33, and (5) 18.5 kBq, 3 µg [^225^Ac]Ac–Macropa–GC33. Animal tumor size, weight, and survival were monitored post-treatment, and animals were sacrificed according to the humane endpoints outlined above. 

### 4.10. Hematological Data Collection and Analysis

Whole blood samples were collected for mice in the TAT study in a maximum 100 µL volume retro-orbitally once weekly (*n* = 5, randomly selected). Blood was separately collected into ethylenediaminetetraacetic acid (EDTA)-lined and lithium heparin-lined collection tubes (Sarstedt, Inc., Newton, NC, USA) and, if not processed immediately after collection, kept overnight in 4 °C and processed once acclimated to room temperature. Blood samples were diluted to meet minimal volume sample requirements for blood analyzer systems. Complete Blood Count (CBC) blood samples collected in EDTA-lined tubes were diluted 1:5 in 1X PBS and analyzed using the Vetscan HM5 (Abaxis, Union City, CA, USA), which was calibrated to accommodate a 1:5 sample dilution using a synthetic whole blood standard (Abaxis, Union City, CA, USA). Basic Metabolic Panel (BMP) blood samples in heparin-lined tubes were diluted 1:3 in 1X PBS, and analyzed using the Vetscan VS2 (Abaxis, Union City, CA, USA) with Comprehensive Diagnostic rotors. 

### 4.11. Biodistribution Study

The biodistribution of 9.25 kBq of [^225^Ac]Ac–GC33 and [^225^Ac]Ac–IgG1 was determined at 48 and 144 h post-injection using HepG2 subcutaneous (right flank) models (female athymic nude mice). Mice with tumor volume below 1500 mm^3^ were selected and divided into GC33 and IgG1 groups for each time point (*n* = 3, except the [^225^Ac]Ac–IgG1 144 h time point where *n* = 2). At 48 and 144 h post-injection, mice were euthanized via CO_2_ asphyxiation. Twelve tissues, including the tumor, were collected. Each sample was weighed and then measured in a gamma counter (2480 Wizard^3^, Perkin Elmer Inc, Shelton, CT, USA) calibrated for an open window (20–2000 kEv). The counts from each sample were decay- and background-corrected, and counts were converted into activity using a calibration curve generated from Ac-225 standards of known activity. The percent injected dose per gram (%IA/g) was estimated by normalizing data to the total activity injected into the corresponding animal and accounting for the mass of each organ. In addition, tumor:blood, tumor:liver, and tumor:muscle ratios were calculated using the mean %IA/g per group. 

### 4.12. Statistical Analysis

Statistical analysis was performed using GraphPad Prism (San Diego, CA, USA). Log-rank Mantel–Cox test was performed to compare survival curves. Tumor volume data were analyzed using a Chi-square test to find statistical significance between the number of animals exceeding 500 mm^3^ in tumor volume. Whole blood and biodistribution analysis were performed using the Student’s *t*-test for unpaired data to identify statistical significance between the mean values of two groups. Statistical significance was defined by *p* < 0.05 (*), 0.01 (**), 0.001 (***), and 0.0001 (****).

## 5. Conclusions

In this radioimmunotherapy study, we demonstrate that targeted alpha particle therapy using the radioconjugate [^225^Ac]Ac–Macropa–GC33 is capable of producing both a modest anti-tumor effect and a clear survival benefit in preclinical models of HCC. However, hematological toxicity remains a significant problem that needs to be addressed.

## Data Availability

Please refer to suggested Data Availability Statements in section “MDPI Research Data Policies” at https://www.mdpi.com/ethics.

## Supplementary Materials

Figure S1: Mass spectrometry results show that GC33 and IgG1 were successfully chelated with Macropa prior to radiolabeling. (**A**). Mass spectrogram demonstrates multiple peaks with molecular weights ranging from 148,182.90 to 155,255.60 g/mol, representing conjugates with 7–12 Macropa per GC33 (148,184 g/mol). Radio instant thin-layer chromatography tracings show excellent radiochemical yields and purity following size-exclusion chromatography of (**B**), [^225^Ac]Ac–Macropa–GC33 and (**C**), [^225^Ac]Ac–Macropa–IgG1. Figure S2: Animals receiving any radioconjugate experienced weight loss following treatment, while tumor burden analysis at day 7 p.i. suggests preferential tumor response. (**A**). Decreases in weight were noted in all groups receiving any radioconjugate. Dashed line denotes starting weight. (**B**). Mean tumor volumes as of day 7 p.i. demonstrate that there is a notable treatment response in all animals receiving either radioconjugate compared to PBS and unmodified GC33 controls. However, a Student’s t-test shows that the 18.5 kBq [^225^Ac]Ac–Macropa–IgG1 treatment group had a larger average tumor volume at 7 days p.i. (425.2 mm^3^, 95% CI: 329.1–521.2) than the 18.5 kBq [^225^Ac]Ac–Macropa–GC33 (318.0 mm^3^, 95% CI: 258.7–377.3) group (*p* = 0.0456). Dashed line denotes the 500 mm^3^ tumor volume threshold used to compare treatment cohorts at 7 days p.i. Statistical significance was defined by *p* < 0.05 (*), 0.01 (**), 0.001 (***), and 0.0001 (****).

## Sample Availability

Samples of the compounds are not available from the authors.
