# Supplementary material for: Glypican-3-Targeted Alpha Particle Therapy for Hepatocellular Carcinoma
Source: Molecules. 2020 Dec 22;26(1):4. doi: 10.3390/molecules26010004 (PMC7792624; doi:10.3390/molecules26010004)
Supplement: Supplementary file 1 [file molecules-26-00004-s001.pdf]

Supplemental figures:

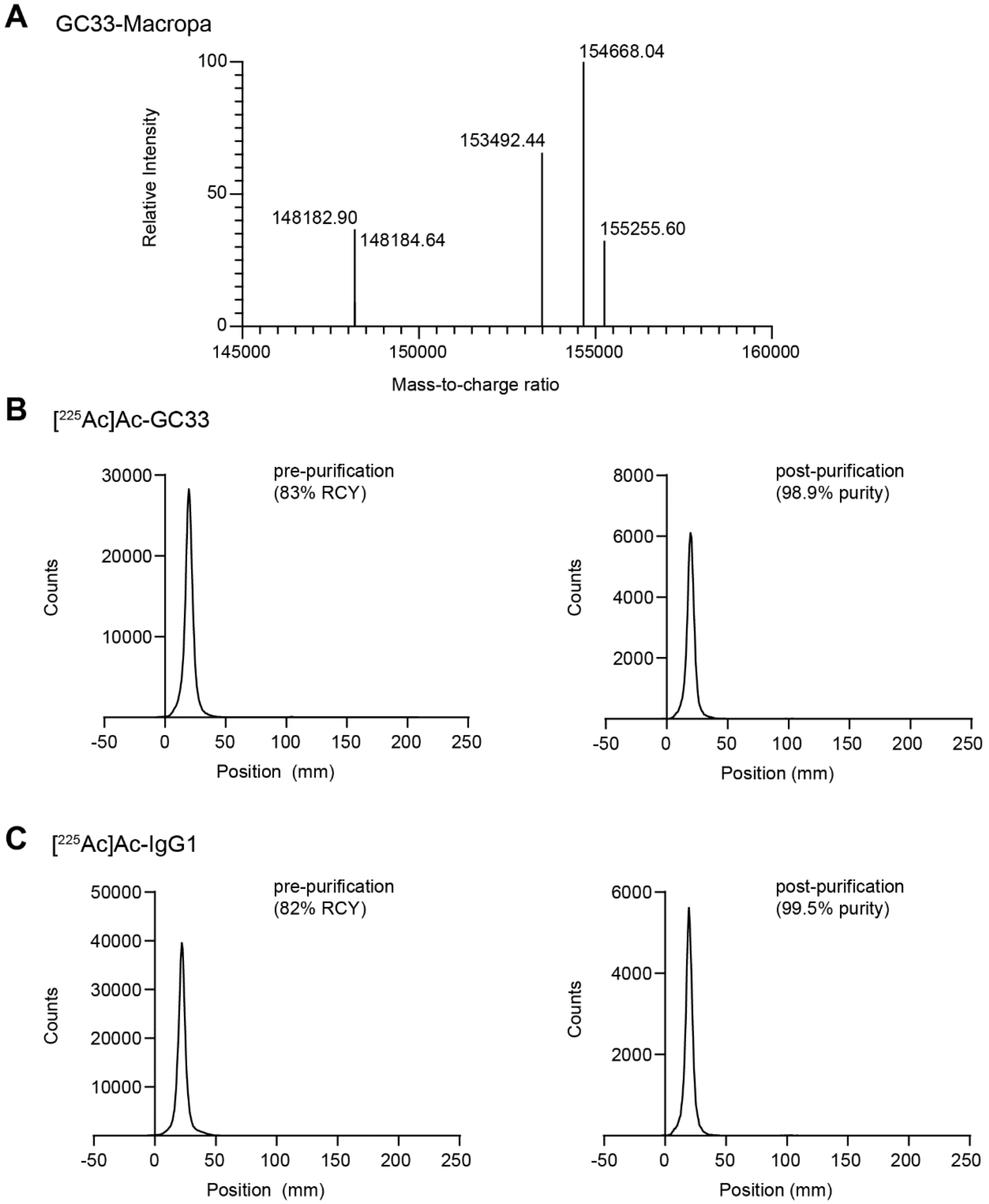

Figure S1. Mass spectrometry results show GC33 and IgG1 were successfully chelated with Macropa prior to radiolabeling. A. Mass spectrogram demonstrates multiple peaks with molecular weights ranging from 148,182.90 g/mol to 155,255.60 g/mol, representing conjugates with 7-12 Macropa per GC33 (148,184 g/mol). Radio instant thin layer chromatography tracings show excellent radiochemical yields and purity following size-exclusion chromatography of **B,** [^{225}Ac]Ac-Macropa-GC33 and **C,** [^{225}Ac]Ac-Macropa-IgG1.

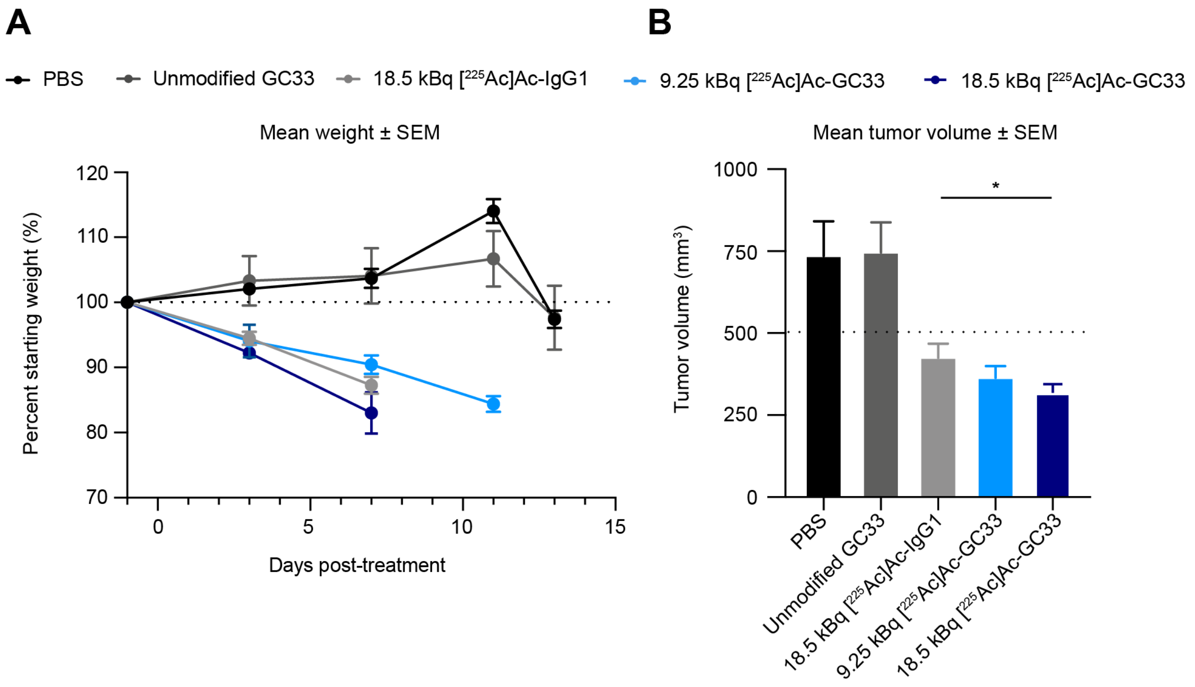

Figure S2. Animals receiving any radioconjugate experienced weight loss following treatment, while tumor burden analysis at day 7 p.i. suggests preferential tumor response. A. Notable decrease in weights were noted in all groups receiving any radioconjugate. Dashed line denotes starting weight. **B.** Mean tumor volumes as of day 7 p.i. demonstrate that there is a notable treatment response in all animals receiving either radioconjugate compared to PBS and unmodified GC33 controls. However, a Student's t-test shows that the 18.5 kBq [^{225}Ac]Ac-Macropa-IgG1 treatment group had larger average tumor volume at 7 days p.i. (425.2 mm^3, 95% CI: 329.1-521.2) than the 18.5 kBq [^{225}Ac]Ac-Macropa-GC33 (318.0 mm^3, 95% CI: 258.7-377.3) group ($p=0.0456$). Dashed line denotes the 500 mm^3 tumor volume threshold used to compare treatment cohorts at 7 days p.i. Statistical significance was defined by $p < 0.05$ (*), 0.01 (**), 0.001 (***), 0.0001 (****).
